# Chitosan-Encapsulated *Coriandrum sativum* Essential Oil Nanoemulsion to Protect Stored Rice Samples Against Fumonisins Contamination and Nutritional Deterioration

**DOI:** 10.3390/foods14223834

**Published:** 2025-11-09

**Authors:** Somenath Das, Sagarika Som

**Affiliations:** Department of Botany, Burdwan Raj College, Purba Bardhaman 713104, West Bengal, India

**Keywords:** *Coriandrum sativum* essential oil, mycotoxin, nanoencapsulation, food preservation

## Abstract

The present study demonstrates encapsulation of *Coriandrum sativum* essential oil in chitosan nanoemulsion and its effectiveness against fungal infestation and fumonisin B_1_ (FB_1_)- and B_2_ (FB_2_)-mediated biodeterioration of stored rice samples. Mycoflora analysis of different rice varieties revealed fungal occurrence and *Fusarium proliferatum*-BRC-R2 as the most toxigenic strain with highest FB_1_- and FB_2_-producing potentiality. GC-MS analysis of *Coriandrum sativum* essential oil (CEO) revealed linalool as the major component. The CEO-loaded chitosan nanoemulsion (Ne-CEO) was characterized by Scanning electron microscopy, X-ray diffractometry, Dynamic light scattering, and Fourier transform infrared spectroscopy. The Ne-CEO showed better antifungal and anti-fumonisin effectiveness as compared to unencapsulated CEO. The antifungal mechanism was associated with reduced ergosterol content, efflux of ions, proteins, nucleic acids, and destruction of plasma membrane integrity. The in silico interaction of linalool with Fum 1 protein confirmed the molecular action of anti-fumonisin activity. Additionally, the Ne-CEO displayed improved antioxidant activity and promising antifungal and anti-fumonisin activity during in situ investigation in rice samples (Gobindobhog variety) along with inhibition of the deterioration of carbohydrate, protein content, and lipid peroxidation without altering organoleptic properties and seed germination potentiality. Overall, the investigation strengthens the potentiality of Ne-CEO as a novel preservative of stored food commodities.

## 1. Introduction

Rice (*Oryza sativa* L.) is one of the most consumed staple cereal foods worldwide, providing 20% of the world’s dietary energy supply [1]. India is among the world’s top five rice producers and consumers. West Bengal is one of the important states in India recognized for maximum cultivation and production of different varieties of rice [2]. Rice grains have been reported as a good substrate for fungal proliferation and mycotoxin contamination in storage conditions [3]. Amongst different mycotoxins, fumonisins secreted by *Fusarium proliferatum* pose the greatest threat to rice grains with compromised nutritional qualities. A number of fumonisin derivatives have been reported; among them fumonisin B_1_ (FB_1_) and fumonisin B_2_ (FB_2_) are common and of the toxic type, causing maximum contamination (70–80%) in cereal-based products [4]. The International Agency for Research on Cancer (IARC) has grouped fumonisins as a class 2B carcinogen in animals and humans [5].

Varieties of chemical fungicides are being used to combat the fungal association and fumonisin synthesis in foods; however, their indiscriminate utilization leads to the development of resistance among fungi and high environmental toxicity [6]. In the current circumstances, *Coriandrum sativum* essential oil (CEO) has been investigated for inhibition of toxigenic fungal species and aflatoxin production. Most importantly, the US Food and drug administration (FDA) and Flavor and Extract Manufacturers Association (FEMA) have approved inclusion of CEO in the “GRAS” category (FEMA No. 2334) for use as flavoring ingredients [7]. However, the practical application of essential oils remains restricted due to some factors like excessive volatility, sensitivity to O_2_, low solubility, and negative effect on organoleptic properties of food commodities [8]. These challenges may be overcome through nanoencapsulation of essential oil into different polymer matrices (polysaccharides, lipids, and proteins). Among different polymers, chitosan (a carbohydrate polymer) is of growing interest in food industries for encapsulation of essential oil owing to its abundance, biodegradability, biocompatible nature, antimicrobial property, and GRAS consideration [9]. Although different methods have been used for encapsulation of essential oils, among them ionic gelation offers great possibility for the development of essential oil-loaded nanoemulsion because of the convenient, economical, non-toxic, and controllable process with a simple operating system [10].

*Coriandrum sativum* L. is an annual aromatic herbaceous plant that belongs to the Apiaceae family and is commonly grown in Asiatic and European countries. The *C. sativum* plant was selected for this study due to its well- documented antimicrobial and antioxidant properties, which are largely attributed to its rich content of essential oils, particularly linalool and other bioactive terpenoids. The essential oil isolated from these fruits possess antifungal, antibacterial, and antioxidant activities [11]. Additionally, *C. sativum* plant is widely available and the dried fruits are approved as spice, medicine, and supplement in pharmaceutical industries by the Flavor and Extract Manufacturers Association, the Council of Europe, and the US Food and drug administration [12]. Previous studies have shown that CEO can inhibit the growth of fungi and reduce the production of aflatoxins [7]. However, the application of CEO for inhibition of fumonisins contamination in rice samples along with enhancement in antifungal efficacy by nanoencapsulation with biochemical and molecular mechanism of action has not been unveiled till now.

The goal of the present research was to assess the antifungal and anti-fumonisin efficacy of CEO and the evaluation of possible improvement in efficacy after encapsulation of CEO in chitosan biopolymer through nanoemulsion preparation. Further, the mechanism of action associated with antifungal and anti-fumonisin effectiveness has also been elucidated. Moreover, the in situ efficacy of Ne-CEO against fungal proliferation and FB_1_ and FB_2_ contamination in storage rice grains along with determination of nutritional contents, lipid peroxidation, organoleptic property evaluation, and phytotoxicity assay was performed for strengthening the recommendation of Ne-CEO as a nano-smart food preservative.

## 2. Materials and Methods

### 2.1. Chemicals and Solvents

Solvents and chemicals viz. chitosan (low molecular weight, 50–160 KDa with deacetylation degree of 75–80%), sodium hypochlorite (NaOCl), potato dextrose agar (PDA), acetonitrile, methanol, chloroform, toluene, tween-20, tween-80, sodium tripolyphosphate (S-TPP), acetic acid, n-hexane, acetone, glacial acetic acid, DPPH, ABTS, potassium persulfate (K_2_S_2_O_8_), petroleum ether, trichloroacetic acid, thiobarbituric acid, sodium hydroxides, sodium sulfate (Na_2_SO_4_), potassium dihydrogen phosphate (KH_2_PO_4_), potassium monohydrogen phosphate (K_2_HPO_4_), benzene, propidium iodide, methyl red, bromocresol green, and hydrochloric acid (HCl) were purchased from Sigma laboratories, Wadala, Mumbai, India and Sisco Research laboratories, Andheri (East), Mumbai, India, with purity grade 97–99%.

### 2.2. Collection of Rice Samples

Different varieties of rice grains viz. Gobindobhog, Boro-minikit, Boro-1153-jath, Barshar-CM, Swarna dhan, and Radhunipagol were collected in between December 2024 and January 2025 from rice fields and authorized rice mills of Purba Bardhaman and adjoining areas of West Bengal, India.

### 2.3. Moisture Content and pH Determination of Rice Samples

To determine the moisture content, 10 g rice samples of different varieties were dried in hot air oven (LuxMed Instruments, New Delhi, India) at 100 °C over a period until the weight remained constant. After that, the moisture content was determined on the basis of difference in fresh weight and dry weight and calculated using the given equation.

$$\mathrm{Moisture}\;\mathrm{content}\;(\%)=\frac{Fresh\;weight\;of\;rice\;samples-Dry\;weight\;of\;rice\;samples}{Fresh\;weight\;of\;rice\;samples}\times 100$$

To determine the pH, 0.5 g of milled rice samples was suspended in 5 mL of deionized water and agitated for 24 h in magnetic stirrer. The pH was measured through an electronic pH meter(Eutech Instruments, Mumbai, India) [13].

### 2.4. Mycobiota Analysis of Rice Samples

Two different methods viz. direct plating and serial dilution were used for mycobiota analysis of different rice samples. For direct plating, surface sterilization of rice samples was performed through 1% sodium hypochlorite solution for 50 s followed by rinsing with deionized water. After that, 4 seeds of different rice samples were placed equidistantly on a Petri plate having 10 mL potato dextrose agar (PDA) medium. During serial dilution, 10 g of milled sample was homogenized into 90 mL of deionized water and five-fold dilutions were developed [14]. After that, 1 mL rice suspension (10^−4^ dilution) was subjected to inoculation in 10 mL PDA medium. Five plates were used for each of the rice varieties and incubated at 25 ± 2 °C for 10 days. The developed fungal colonies were regularly examined, and fungal species were identified through morphological and cultural characteristics. The relative density and occurrence frequency of different fungi in rice samples were calculated by the following formula:

$$\mathrm{Relative}\;\mathrm{density}\;(\%)\;=\;\frac{Number\;of\;colonies\;of\;each\;fungus}{Total\;number\;of\;colonies\;of\;all\;fungal\;species}\times 100$$

$$\mathrm{Occurrence}\;\mathrm{frequency}\;(\%)=\frac{Number\;of\;fungal\;isolates\;on\;each\;sample}{Total\;number\;of\;fungal\;isolates\;on\;all\;rice\;samples}\times 100$$

### 2.5. Detection of Toxigenic Strain of F. proliferatum

FB_1_- and FB_2_-producing potentiality of *F. proliferatum* isolates from different varieties of rice samples were assessed by the method of Singh et al. [15]. At first, the fungal isolates were incubated in PDA medium in Petri plates for 5 days at 20 ± 2 °C. After that, the plates were left in refrigerator (4 °C) for 12 days. Subsequently, the mycelial growth on agar medium (1 cm piece) was added to 96% methanol (50 mL), followed by shaking at 200× *g* for 24 h and being subjected to filtration by sterile muslin cloth. Washing of the residue was performed through methanol and it was evaporated at ambient temperature. Thin layer chromatography (TLC) method was applied to determine FB_1_ and FB_2_ content. After that, 10 µL of the extract was spotted onto TLC plate made up of silica gel G together with standard (5 µg FB_1_ and FB_2_). p-anisaldehyde spraying was performed to view the TLC plate under 254 nm and 365 nm UV irradiation. Bluish-green spots of FB_1_ and FB_2_ were observed with R_f_ value 0.25 and 0.30, respectively.

### 2.6. Extraction and Chemical Profiling of Coriandrum sativum Essential Oil (CEO)

Briefly, 500 g of dried *Coriandrum sativum* fruits was poured for hydrodistillation in Clevenger apparatus (Borosil Pvt. Ltd., Mumbai, India). An electro mantle was used as the heating source, and the reactor volume was about 2.5 L, where dried fruits along with 3 L of distilled water were maintained. The extraction process was extended to about 4 h and operated at power of 350 W. The condensate containing CEO and water was dehydrated over Na_2_SO_4_ for removal of excess water content and the CEO was stored at 4 °C.

$$\mathrm{Yield}\;\mathrm{of}\;\mathrm{CEO}=\frac{Amount\;of\;extracted\;essential\;oil\;(mL)}{Amount\;of\;dried\;fruits\;used\;(kg)}$$

Chemical profiling of CEO was carried out by HP-5890 Series II Gas-Chromatograph (Hewlett-Packard, Palo Alto, CA, USA) associated with HP-5 column (30 m × 0.25 mm × 0.25 µm). Different conditions for GC were temperature at injection site and ion source 250 °C, temperature of column isothermal at 70 °C (2 min) followed by programmed increase up to 250 °C, and carrier gas was helium with constant flow of 1 mL/min. Injection of 1 µL of CEO (acetone diluted) was performed on the column. The GC column was attached to MS and spectra were obtained at 70 eV ionization in the EI mode. The mass analyzer was scanned between 40 and 500 amu. Compounds were identified on the basis of retention indices using a homologous series of C_9_−C_28_ n-alkane (Sigma-Aldrich, St. Louis, MO, USA) and the spectral data was compared with NIST (2011) libraries [16].

### 2.7. Synthesis of CEO-Loaded Chitosan Nanoemulsion (Ne-CEO)

Ne-CEO was developed by ionic gelation protocol of Hosseini et al. [17] with slight modification. Chitosan solution (1.25%; *w*/*v*) was prepared in 1% (*v*/*v*) aqueous acetic acid and stirred at ambient temperature for 8–10 h. Thereafter, 0.27 g tween-80 was dispersed into chitosan solution and agitated for 1 h. Required volumes of CEO were mixed with tween-20 and dropped gradually to chitosan solution at the time of homogenization (IKA Works, Staufen, Germany) (12,550× *g* for 12 min, 6 °C) with resultant formation of different ratios (*w*/*v*) (1:0.0, 1:0.2, 1:0.4, 1:0.6, 1:0.8, and 1:1) of chitosan to CEO emulsion. Thereafter, sodium tripolyphosphate (S-TPP, 0.4%) was added to homogenized emulsion of chitosan with continuous stirring (40 min) over magnetic stirrer to facilitate cross-linking at 25 ± 2 °C. After that, centrifugation of emulsion was performed at 11,285× *g* for 12 min, and the collected pellet was mixed with 10 mL of ultrapure water followed by sonication (Branson ultrasonics, Danbury, CT, USA) (ice bath condition) for 4 min (0.7 s pulse on and 0.3 s pulse off). Chitosan nanoemulsion (Ne-Cs) was prepared following the same procedure without addition of CEO.

### 2.8. Encapsulation Potency (EP), Loading Potency (LP), and Encapsulation Yield (EY) of Ne-CEO

For this, 300 µL of Ne-CEO was suspended into 3 mL of n-hexane and centrifuged at 12,500× *g* for 13 min (4 °C). Thereafter, the upper transparent layer was sucked into an eppendorf tube, and optical density was noted at λ_max_ 278 nm. CEO loading in chitosan nanoemulsion was determined according to the calibration curve of CEO (Y = 0.0273 X + 0.0079). The given formula was utilized to calculate % EP, LP, and EY of Ne-CEO.

$$\mathrm{EP}\;(\%)=\frac{Total\;amount\;of\;CEO\;encapsulated\;into\;chitosan\;nanoemulsion}{Weight\;of\;nanoemulsion}\times 100$$

$$\mathrm{LP}\;(\%)=\frac{Total\;amount\;of\;CEO\;encapsulated\;into\;chitosan\;nanoemulsion}{Initial\;amount\;of\;CEO}\times 100$$

$$\mathrm{EY}\;(\%)=\frac{Amount\;of\;total\;nanoemulsion\;synthesized}{Sum\;total\;of\;all\;the\;individual\;componnets}\times 100$$

### 2.9. Characterization of Ne-CEO

#### 2.9.1. Analysis of Particle Size, Polydispersity Index (PDI), and Zeta Potential

Size of particles, PDI, and zeta potential of Ne-Cs and Ne-CEO was measured through ZEN-3600 Zeta potentiometer (Malvern Instruments, Great Malvern, UK) after dilution of 1 μL of sample to 5 mL of ultrapure water with scattered light fixed angle 90°.

#### 2.9.2. Analysis Through Scanning Electron Microscopy (SEM)

For this examination, 2 drops of Ne-Cs and Ne-CEO were taken on cover slip and desiccated in sterilized air pursued by gold coating of the film under high vacuum (E-1010, Hitachi, Tokyo, Japan). Then, the samples were subjected to SEM (Evo-18 researcher, Zeiss, Jena, Germany) analysis.

#### 2.9.3. Fourier Transform Infrared Spectroscopy (FTIR) Analysis

FTIR of CEO, chitosan, Ne-Cs, and Ne-FEO was performed in Nicolet-5700 Fourier transform infrared spectroscopy (Thermo Fisher Scientific, Madison, WI, USA). The samples were scanned between 500 and 4000 cm^−1^ wave number with 32 scans and 5.0 cm^−1^ resolution.

#### 2.9.4. X-Ray Diffractometry (XRD) Analysis

XRD assay of chitosan, Ne-Cs, and Ne-CEO was performed in X-R diffractometer (D-5000, Karlsruhe, Germany) in between the 2θ range of 5–60° and 0.05 °/min speed angle.

### 2.10. Release of CEO

Release of CEO was performed in ethanol-mixed phosphate-buffered saline (PBS, pH 7.0) on the basis of the methodology provided by Amiri et al. [18]. For this, 700 µL of Ne-CEO was centrifuged at 9523× *g* for 16 min (4 °C). Thereafter, water was poured and 1.2 mL of PBS and ethanol mixture was added into an eppendorf tube containing particles. The suspension was vortex-mixed and incubated for 7 days at ambient temperature. At definite time intervals, centrifugation of the samples was performed and 100 µL of samples were homogenized into 3 mL ethanol-mixed PBS, succeeded by recording of absorbance at 288 nm. Each and every time, the equivalent amount of fresh PBS was replaced. CEO release was quantified by the given equation.

$$\mathrm{CEO}\;\mathrm{release}\;(\%)=\sum_{t=0}^{t}\frac{Accumulative\;amount\;of\;CEO\;release\;at\;each\;sampling\;time}{Initial\;amount\;of\;CEO\;loaded\;in\;nanoemulsion}\times 100$$

### 2.11. Antifungal and Anti-Fumonisin Effectiveness of CEO and Ne-CEO: In Vitro Study

Required concentrations of CEO (0.1–0.7 µL/mL) and Ne-CEO (0.1–0.4 µL/mL) were dissolved into 10 mL PDA medium and 10 µL FP-BRC-R2 spore suspension was added. Control consisted of PDA medium with only FP-BRC-R2 spore suspension. Control and treatment sets were subjected to incubation at 25 ± 2 °C for 10 days. Minimum concentration of CEO and Ne-CEO representing complete suppression of FP-BRC-R2 growth were reported as minimum inhibitory concentrations (MICs). Fungitoxic analysis of CEO and Ne-CEO against *F. verticillioides*, *Aspergillus flavus*, *A. niger*, *A. repens*, *Penicillium italicum*, *Alternaria alternata*, *A. humicola*, and *Curvularia lunata* was conducted in PDA medium at the MIC by inoculating the above-mentioned fungal disk (5 mm) [19], and inhibition of growth was calculated by the given equation.

$$\%\mathrm{Inhibition}=\frac{Inhibition\;of\;fungal\;colony\;in\;control-Ingibition\;of\;fungal\;colony\;in\;treatment}{Inhibition\;of\;fungal\;colony\;in\;control}\times 100$$

To determine the FB_1_ and FB_2_ content in the control and treatment sets, the process described in Section 2.5 was used.

### 2.12. The Mechanisms Related to Antifungal and Anti-Fumonisin Activity

#### 2.12.1. Effect on Ergosterol Synthesis

The method of Sun et al. [20] was utilized for the ergosterol determination in FP-BRC-R2 cells. The fungal biomass fumigated with CEO and Ne-CEO was subjected to mixing with 15% NaOH solution and 90% ethanol followed by evaporation at water bath at 75 °C for 1 h. Extraction of sterol was performed by addition of 5 mL of benzene and evaporated under a stream of N_2_. The absorbance of ergosterol and 24,28 dehydroergosterol was determined at 282 and 230 nm, respectively. The below-mentioned equation was used for the calculation of ergosterol content.

$$\%\;\mathrm{Ergosterol}=\frac{100}{1+\frac{Optical\;density\;at\;230\;nm}{Optical\;density\;at\;282\;nm}\times 0.56}$$

% 24,28 dehydroergosterol = 100% − % Ergosterol

#### 2.12.2. Effect on Leakage of Ions, 260 nm, and 280 nm Absorbing Materials

For this, the 10-days cultured biomass of FP-BRC-R2 cells was washed with sterile deionized water, suspended in 0.85% NaCl solution (20 mL), and incubated with CEO and Ne-CEO at 25 ± 2 °C for 12 h. After filtering the biomass by sterile muslin cloth, leakage of Mg^2+^, K^+^, and Ca^2+^ ions from FP-BRC-R2 cells was determined by Atomic absorption spectrophotometry (PerkinElmer, Waltham, MA, USA). The filtrate was also used for the determination of nucleic acids and protein leakage by measuring the optical density at 260 nm and 280 nm through UV–visible spectrophotometry (Lasany International, Panchkula, India) [21].

#### 2.12.3. Integrity of Plasma Membrane

The plasma membrane integrity of FP-BRC-R2 cells was determined through propidium iodide (PI)-binding protocol of Liu et al. [22] with little modification. To achieve this, 200 mg of 10-days-grown FP-BRC-R2 biomass was treated with different concentrations of CEO and Ne-CEO followed by incubation at 25 ± 2 °C for 12 h. After that, the biomass was cleaned with PBS and stained in PI dye (10 µg/mL) for 5 min. Eventually the biomass was again rinsed with PBS, crushed, and centrifuged at 5000× *g* for 10 min. The supernatant was analyzed through fluorescence spectrophotometry (Shimadzu Corporation, Kyoto, Japan) at excitation and emission wavelengths of 546 and 590 nm, respectively.

#### 2.12.4. Molecular Modeling: In Silico Anti-Fumonisin Potency

Structure of linalool in SDF format was retrieved from PubChem. Uniprot was used to download Fum 1 protein sequence in FASTA format. The three-dimensional structure of Fum 1 protein was developed through PHYRE 2 protein recognition server. For determination of the comprehensive molecular interaction of linalool with Fum 1, PatchDock algorithm-based complementarity tool was utilized. After that, the best 20 models were screened with the help of FireDock system based on global energy, atomic contact energy (ACE), attractive Van der Waals force, and repulsive Van der Waals force. Selection of optimized interaction between linalool and Fum 1 was based on the energetically optimized state and determined through Chimera 1.8.1. software [23].

### 2.13. Antioxidant Activity of CEO and Ne-CEO

#### 2.13.1. DPPH (2,2-Diphenyl-1-Picrylhydrazyl) Assay

Different concentrations of CEO and Ne-CEO were added to 4 mL DPPH solution (0.1 mM). After that, the mixture was subjected to incubation at 25 ± 2 °C for 30 min and optical density was noted at 517 nm by UV–visible spectrophotometer [24]. The radical neutralization potency was measured by the below-mentioned equation.

$$\%\;\mathrm{Radical}\;\mathrm{neutralization}\;\mathrm{potency}=\frac{Absorbance\;of\;Blank-Absorbance\;of\;Sample}{Absorbance\;of\;Blank}\times 100$$

#### 2.13.2. ABTS (2,2′-Azinobis-3-Ethylbenzothiazoline-6-Sulfonic Acid) Assay

The methodology of Re et al. [25] with little modification was used for the ABTS assay. The ABTS radical cation was produced by mixing ABTS stock solution (7 mM) with 2.45 mM potassium persulfate in a stoichiomatric ratio of 1:0.5 and allowing the mixture to incubate in a dark room for 8 h at 25 ± 2 °C. After that, adjustment of the optical density value of ABTS radical solution was maintained to 0.70 ± 0.05 at 734 nm by addition of 80% ethanol. Subsequently, various concentrations of CEO and Ne-CEO were mixed with 2 mL of ABTS solution and optical density was observed at 734 nm after 6 min of dark incubation. The radical neutralization potency was calculated by the same formula as provided by the DPPH assay.

### 2.14. Evaluation of In Situ Antifungal and Anti-Fumonisin Activity of CEO and Ne-CEO in Stored Rice

The in situ potency of CEO and Ne-CEO was performed in rice samples (Gobindobhog variety) using the protocol of Kedia et al. [26]. Prior to experiment, the rice samples were cleaned with distilled water followed by surface sterilization with 1% NaOCl solution to remove the surface contaminants. Different sets were developed for the conduction of the experiment.

(i) Uninoculated control (UIC).
(ii) Inoculated control (IC).
(iii) Uninoculated treatment with CEO (UI-t-CEO).
(iv) Inoculated treatment with CEO (I-t-CEO).
(v) Uninoculated treatment with Ne-CEO (UI-t-Ne-CEO).
(vi) Inoculated treatment with Ne-CEO (I-t-Ne-CEO).

To conduct the experiment, 1 kg of rice samples were placed separately in different glass containers (2.5 L). In case of inoculated control and treatment sets, 1 mL of FP-BRC-R2 spore suspension was introduced to the rice samples by spraying; however, the uninoculated control and treatment sets were not inoculated with FP-BRC-R2 spore suspension. The rice samples were fumigated with MIC dose of CEO (0.7 µL/mL) and Ne-CEO (0.4 µL/mL). Requisite amounts of CEO and Ne-CEO were soaked separately in cotton swab according to the aerial volume of the glass container and attached to the lid of container. The containers were kept airtight followed by storage for 6 months at 25 ± 2 °C (75% relative humidity). After completing the storage period, mycobiota analysis of rice samples was performed by serial dilution method as described in Section 2.4. Colonies of FP-BRC-R2 were counted after 10 days. The below-mentioned formula was used for determination of the percent protection of rice samples.

$$\%\;\mathrm{Protection}=\frac{Total\;no.of\;FP-BRC-R2\;colony\;in\;control\;set-Total\;no.of\;FP-BRC-R2\;colony\;in\;treatment\;set}{Total\;no.of\;FP-BRC-R2\;colony\;in\;control\;set}\times 100$$

To determine the FB_1_ and FB_2_ content, 20 g of milled rice was mixed with AMW mixture (25 mL acetonitrile + 25 mL methanol + 50 mL water), followed by stirring for 30 min. After that the mixture was filtered and filtrate was mixed with PBS (25 mL), followed by further purification through column chromatography with rate of flow at 1.5 drop/s. Further, the eluted sample was dried by N_2_ stream, reconstituted in 2 mL acetonitrile–water mixture (30:70; *v*/*v*), and finally prepared for HPLC analysis. After that, 5 µL of the sample was injected into C18 HPLC column (150 × 4.6 mm^2^, 5 µm) (Waters India Pvt Ltd, Bengaluru, India) and analyzed by comparison with standard curve of FB_1_ and FB_2_. Detection was performed using Prostar 363 module with software version 6.20. The mobile phase consisted of GAW mixture (1 mL glacial acetic acid + 30 mL acetonitrile + 69 mL water) with 1 mL/min flow rate. Preparation of FB_1_ and FB_2_ stock solution was performed in 50 mL water and 50 mL acetonitrile. To develop the HPLC standard, the requisite volume of stock solution was dried and reconstituted in water and acetonitrile (30:70; *v*/*v*). The linearity of analytical response was assessed using calibration curve in the concentration of 0.1–1.0 ng/µL for FB_1_ and FB_2_, and results were expressed in µg/kg of rice samples [15].

### 2.15. Effect of CEO and Ne-CEO on Carbohydrate, Protein Content, and Lipid Peroxidation in Rice

The protocol of Albalasmeh et al. [27] was utilized for carbohydrate estimation in different rice samples. The optical density of color was measured at 490 nm by UV–visible spectrophotometer. The Kjeldahl method of Barbano et al. [28] was utilized for protein content evaluation in rice samples. Determination of nitrogen content was performed by HCl-mediated titration through 1 mg/mL methyl red-mixed ethanol and bromocresol green. Peroxidation of rice lipids was evaluated on the basis of TBARS method given by Das et al. [19]. The extinction coefficient of malondialdehyde (MDA) was 155,000 M^−1^cm^−1^ and MDA content in rice samples was presented as µM/gFW.

### 2.16. Effect of CEO and Ne-CEO on Organoleptic Properties of Rice

Organoleptic property was performed in rice sample after 6 months of storage by five-point hedonic scales using the method of Prasad et al. [29]. Ten different participants (aged between 32 and 45 years of both sexes) belonging to the academic community of our institution were involved in the evaluation process. The evaluation and informed consent forms were given to each of the participants (permitted by Research and Development division of Burdwan Raj College, (BRC-2025/01)) along with rice samples coded with numbers 1–5. The rice samples were subjected to cooking without pressure for half an hour and served in disposable containers. Evaluation was performed on the basis of texture, color, flavor, odor, and overall acceptance of the rice samples. We have followed the declaration of Helsinki and the Medical Association ethics to involve the human participants. Two minutes were given to each of the participants to assess the texture, color, flavor, and odor of rice and 1 min of rest. The participants were asked to express their assessment of the quality of rice samples using five different numbers: 1 = Quality with very bad nature, 2 = Quality with bad nature, 3 = Regular, 4 = Quality with good nature, and 5 = Quality with very good nature.

### 2.17. Phytotoxicity Assay of Rice Seeds

Phytotoxicity assay of rice seeds (control, CEO, and Ne-CEO fumigated) was performed in terms of the germination of plumule and radicle following the method of Shukla et al. [30]. After 6 months of storage, different sets of rice samples were taken and 5 seeds were randomly collected from different sets, followed by equidistant placing at the moistened filter paper on the bottom of the Petri plate. After that, incubation of the Petri plates was carried out at 25 ± 2 °C and length of plumule and radicle were measured through centimeter scale at different time intervals.

### 2.18. Statistical Analysis

Each and every experiment was carried out in triplicate sets and results were expressed as mean ± standard error. Data were subjected to one-way analysis of variance (ANOVA). Means were separated by Tukey’s multiple comparison tests when ANOVA was significant (*p* < 0.05).

## 3. Results and Discussions

### 3.1. Moisture Content, pH, and Mycobiota Analysis of Rice Samples

The fungal infestation in rice samples is governed by different abiotic factors like moisture content and pH. The moisture content of six different rice varieties ranged between 9.85 ± 0.17–11.96 ± 0.18% (*p* < 0.05, df = 5, 12, F = 19.91) (Table 1) which has been considered as conducive range for fungal association [31]. Kana et al. [32] reported high moisture content in association with little aeration and high relative humidity accelerate the fungal proliferation and mycotoxin synthesis. pH of different rice samples was found in 6.08 ± 0.92–6.87 ± 0.18 (*p* < 0.05, df = 5, 12, F = 7.238) (Table 1), which is favorable for growth of different storage fungi [31]. In addition to pH and moisture content, the chemical profile of rice is also responsible for fungal association and fumonisin contamination. Mycobiota analysis of different rice samples showed nine different fungal species viz. *Fusarium proliferatum*, *F. verticillioides*, *Aspergillus flavus*, *A. niger*, *A. repens*, *Penicillium italicum*, *Alternaria alternata*, *A. humicola*, and *Curvularia lunata*. Relative density of different fungi in rice samples ranged between 1.97 and 32.20% and gobindobhog rice variety showed the highest occurrence frequency (22.59%) among six different rice varieties tested for the investigation (Table 2). Quantification of FB_1_ and FB_2_ content of various isolates of *F. proliferatum* from different varieties of rice samples was performed through TLC and FP-BRC-R2 displayed maximum FB_1_ (142.20 µg/L) and FB_2_ (135.22 µg/L) content isolated from gobindobhog variety (Table 3). Hence, the result of mycobiota analysis emphasized that rice samples have been heavily biodeteriorated by different storage fungi, especially *F. proliferatum* and their associated fumonisins contamination.

### 3.2. Extraction and Chemical Profiling of CEO

**Figure 1:**
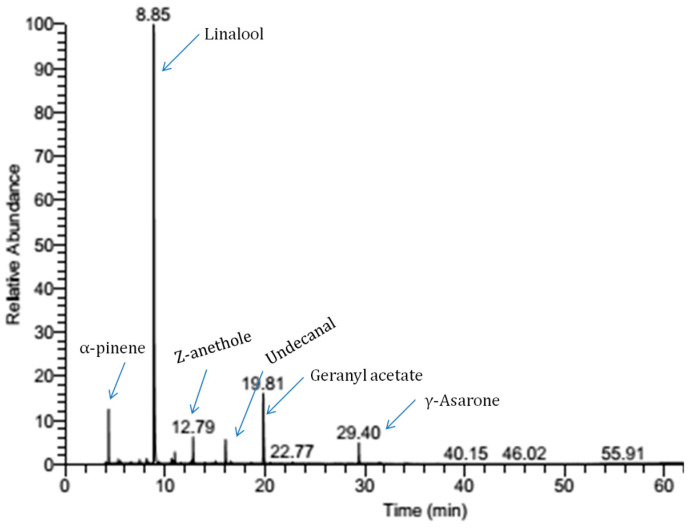
GC-MS chromatogram of CEO.

The CEO was isolated from dried fruits of *Coriandrum sativum*. The yield of CEO through hydrodistillation was 5 mL/kg of fruits. GC-MS profiling of CEO revealed 15 different compounds; among them, linalool (66.46%) and geranyl acetate (14.22%) were identified as the major compounds (Figure 1, Table 4). A recent investigation of Nouioura et al. [33] suggested linalool as the principal compound of *Coriandrum sativum* essential oil. The study of Viuda-Martos et al. [34] reported geranyl acetate as the major ingredient of Indian coriander fruits. Different factors like endogenous and exogenous elements are associated with physiological and anatomical properties of plants resulting in variations in biosynthesis pathways of volatiles [35]. The essential oil synthesis pathway may exhibit variation on the basis of seasonal condition, plant tissue, chemotype, and ecotype alteration, as well as DNA adaptation [36,37]. Therefore, chemical profiling of essential oil is important before nanoencapsulation-based practical application in food commodities.

### 3.3. Synthesis and EP, LP, and EY of Ne-CEO

The CEO was entrapped into chitosan nanobiopolymer following the two-step process of droplet formation and solidification [38]. The affinity of amino groups of chitosan with phosphate groups of S-TPP along with other functional groups of CEO resulted in the formation of nanoemulsion. Tween-80 helped to stabilize the emulsionic droplets into chitosan polymer [39]. Different ratio of chitosan/CEO (*w*/*v*) was constructed to select the nanoemulsion system with maximum encapsulation and loading potency.

The EP and LP are quantitative indicators to indicate the loading of active constituents in chitosan nanomatrix and also determine the protection of components against external oxidation [40]. The EP and LP of different chitosan-to-CEO (*w*/*v*) ratios ranged between 29.63 and 92.11%, and 0.82 and 5.04%, respectively (Table 5). The EP and LP were found to increase up to 1:0.8 the ratio (*w*/*v*) of chitosan to CEO, whereas, at higher concentrations (1:1 ratio, *w*/*v*), they were considerably decreased. Decrease in encapsulation and loading potency at 1:1 ratio of chitosan/CEO (*w*/*v*) has been attributed to the less efficient dispersive forces during homogenization of particles and increased tendency of oil vesicles to coalesce each other. At this time, the amount of chitosan polymer might not be sufficient for encapsulation of all oil vesicles. The probability of the existence of unencapsulated oil vesicles became higher. The loss of these oil vesicles during isolation may reduce the encapsulation and loading potency [41]. Some other factors like hydrophilicity, degree of deacetylation, and molecular weight of chitosan are also pointed out as a critical factor for lowering the LP and EP values at higher chitosan/CEO ratio [42]. Our result is consistent with the investigation of Singh et al. [43], suggesting reduction in % EP and LP of *Aniba rosaeodora* essential oil-loaded chitosan nanoparticle at 1:1 ratio. EY of chitosan to CEO at 1:0.8 (*w*/*v*) ratio was noted as 27.96 ± 3.01%, indicating maximum entrapment of CEO into chitosan polymer. As the highest EP and LP was achieved at 1:0.8 (*w*/*v*) chitosan-to-CEO ratio, only this ratio of nanoemulsion was used for determination of encapsulation yield as well as further characterization and biological testing.

### 3.4. Physico-Chemical Characterization of Ne-CEO

#### 3.4.1. Particle Size, Zeta Potential, and Polydispersity Index

**Figure 2:**
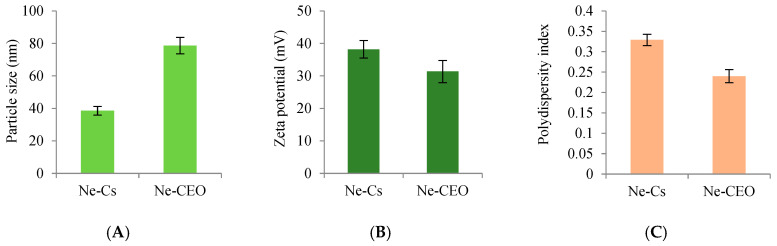
(**A**) Particle size of Ne-Cs and Ne-CEO [F (1, 4) = 102.32, *p* < 0.05], (**B**) zeta potential of Ne-Cs and Ne-CEO [F (1, 4) = 9.147, *p* < 0.05], (**C**) polydispersity index of Ne-Cs and Ne-CEO [F (1, 4) = 58.06, *p* < 0.05]. Note: Values are mean (n = 3) ± SE.

Average size of Ne-Cs and Ne-CEO particles was 38.54 and 78.67 nm, respectively (Figure 2A). A significant increase in size of Ne-CEO particles was observed due to loading of CEO in chitosan biopolymer. Our result shows conformity with the finding of Jiang et al. [44] regarding increased size of *Eucommia ulmoides* essential oil-loaded chitosan particles from 200.60 to 276 nm. The zeta potential value of Ne-Cs (+38.20 mV) was higher than Ne-CEO (+31.37 mV) (Figure 2B), which demonstrated that the entrapment of CEO led to scattering of nanoemulsionic particles in water. The adsorption or scattering of essential oil droplets on the chitosan nanoparticle surface may lead to enhanced exposure of protonated amine groups and reduced shielding by counter-ions, thereby increasing the net positive surface charge. Remarkable decrement in zeta potential value of Ne-CEO reflected CEO adsorption at the exterior portion of chitosan particles which led to concealing the free amino groups of chitosan [45]. The value of zeta potential ± 30 mV validates the stability of the system. A comparable decrease in the zeta potential value of *Cinnamomum zeylanicum* essential oil-loaded chitosan nanoparticles was demonstrated by Mohammadi et al. [46]. PDI is a measure for degree of uniformity of particle distribution in emulsion system. Low PDI value of Ne-CEO as compared to Ne-Cs (Figure 2C) suggested the narrow size and uniform distribution of emulsionic particles. The current result is in accordance with Samling et al. [47], displaying a good uniformity and dispersion of *Cyanometra cauliflora* essential oil-entrapped chitosan nanoparticles. Hence, small particle size with uniform distribution and maximum stability facilitate the utilization of Ne-CEO as innovative smart and green candidates to protect rice grains from fungal infestation and fumonisins contamination.

#### 3.4.2. SEM Observation

**Figure 3:**
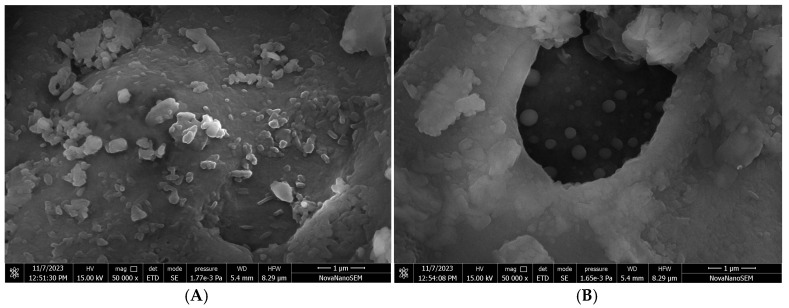
(**A**) Scanning electron microscopic (SEM) image of Ne-Cs; (**B**) SEM image of Ne-CEO.

SEM images presented ovoid-globular to elongated microstructures with smooth-walled surface. The Ne-Cs particles ranged between 24.53 and 41.20 nm, while Ne-CEO particles displayed larger diameters in between the range of 50.79 and 85.27 nm (Figure 3A,B), which resulted due to encapsulation of CEO as core material into chitosan polymer. The current resulting particle size is similar to the zeta sizer assay; however, some size variation was observed which has been attributed to the sensitivity of different instruments. Higher particle size of Ne-CEO by SEM analysis was due to particle aggregation, whereas in DLS analysis finely diluted particles were used, hence avoiding the agglomeration of particles [48]. The finding is in agreement with the investigation of Cai et al. [49] regarding increment in chitosan particle size after incorporation of *Ocimum basilicum* essential oil.

#### 3.4.3. FTIR Analysis

**Figure 4:**
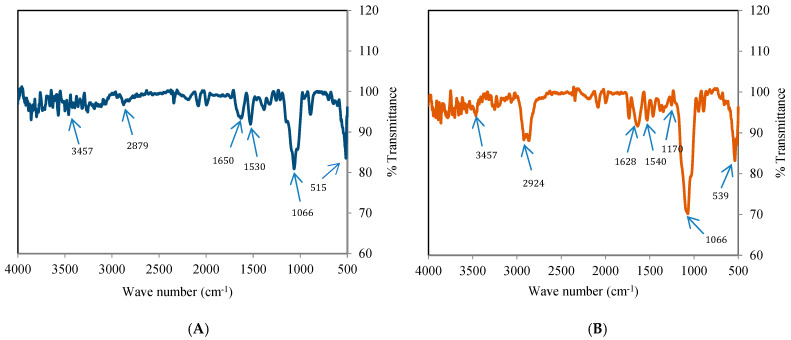

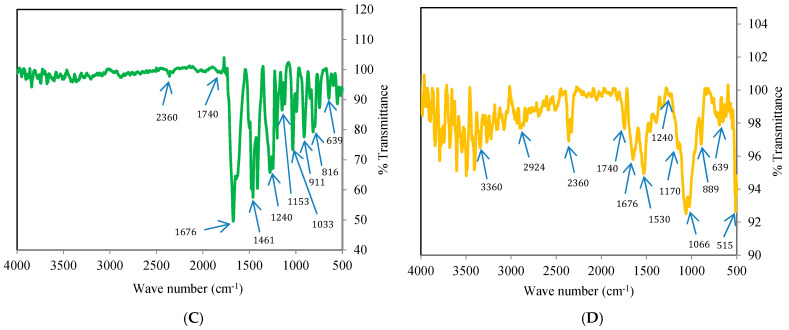
(**A**) Fourier transform infrared (FTIR) spectrum of chitosan, (**B**) FTIR spectrum of Ne-Cs, (**C**) FTIR spectrum of CEO, (**D**) FTIR spectrum of Ne-CEO.

Chitosan displayed various peaks at 3457 (NH_2_ and –OH group bending), 2879 (symmetrical stretching of –CH_2_ group and vibrational modes of –CH_3_ group), 1650 (amide I, C=O stretching), 1530 (N-H bending), 1066 (symmetrical stretching of C-O-C), and 515 (in-plane bending of C-H) cm^−1^ (Figure 4A). In Ne-Cs the amide peaks showed reduction in intensity and shifting of N-H bending peak from 1530 to 1540, and amide I peak from 1650 to 1628 cm^−1^ (Figure 4B). Most importantly, the development of a phosphate-related peak at 1170 cm^−1^ confirmed the electrostatic binding of NH_3_^+^ and PO_4_^3−^ groups [50]. The peak of chitosan at 2879 was slightly relocated to 2924 cm^−1^ in Ne-Cs. The CEO showed different peaks at 2360 (C-H stretching), 1740 (C=O stretching), 1676 (aromatic ring bending), 1461 (C=C bending), 1240 (C-H vibration absorption of benzene ring), 1153 (C=O stretching), 1033, 911 (-OH bending), 816 (C-N stretching), and 639 (stretching ring of oxygen) cm^−1^ reflecting the presence of different compounds [51] (Figure 4C). Additional shifting as well as occurrence of corresponding CEO peaks was observed in Ne-CEO (Figure 4D), confirming successful molecular interaction of CEO compounds with chitosan chains rather than a simple physical entrapment.

#### 3.4.4. XRD Analysis

**Figure 5:**
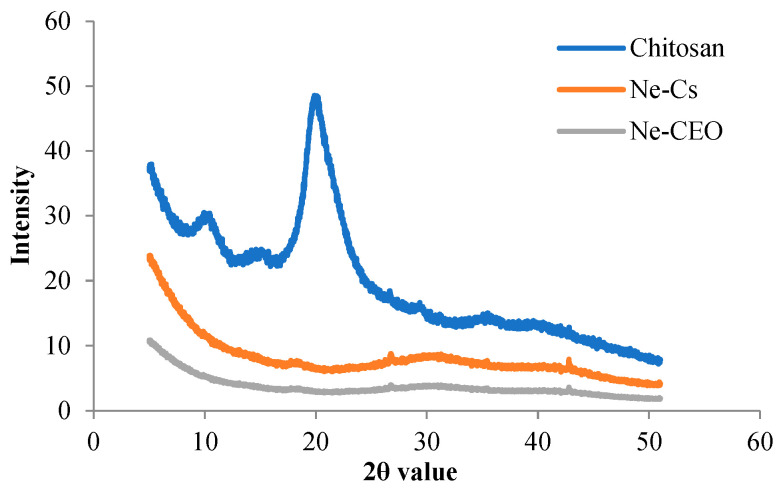
X-ray diffraction (XRD) analysis of chitosan, Ne-Cs, Ne-CEO.

The present investigation revealed highly crystallized structure of chitosan powder with a sharp peak of 2θ value at around 20° (Figure 5). In Ne-Cs, the sharp peak has almost disappeared with an amorphous structure, which could be associated with destruction of chitosan crystallinity by cross-linking during interaction of NH_3_^+^ group of chitosan with PO_4_^3−^ group of S-TPP [52]. However, after entrapment of CEO into chitosan nanoemulsion, the peak intensity became narrower and completely destructed the complex structural organization of chitosan (Figure 5). The result is consistent with the finding of Song et al. [53], illustrating the loss in degree of crystallinity of chitosan nanoparticle following the addition of *Citrus reticulata* essential oil.

### 3.5. In Vitro Release of Ne-CEO

**Figure 6:**
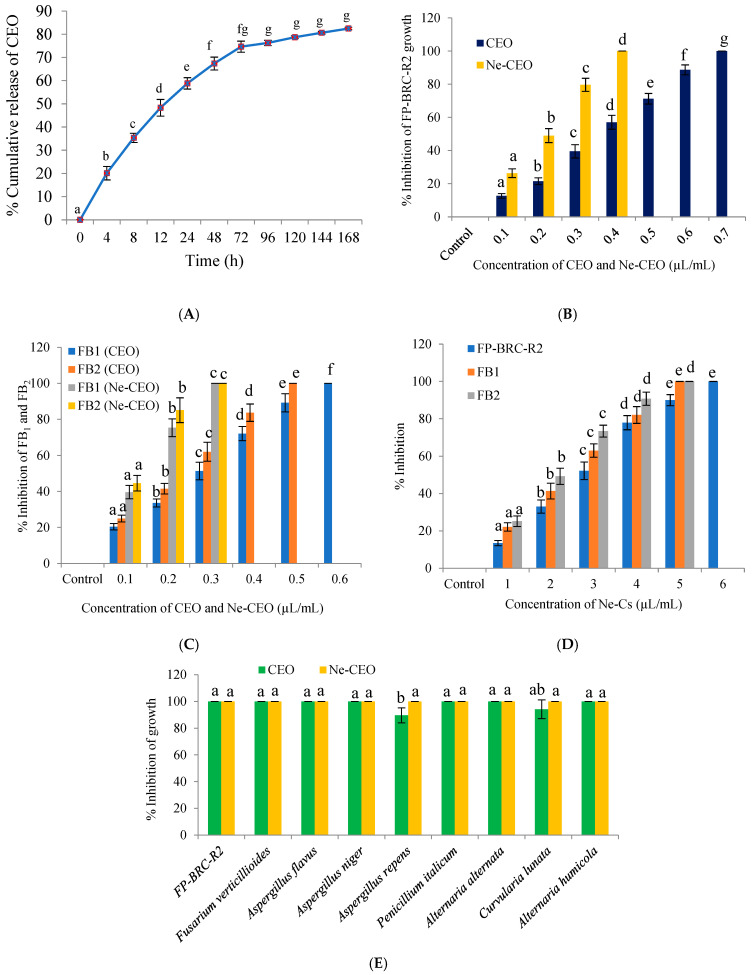
(**A**) In vitro release of CEO [F (10, 22) = 214.71, *p* < 0.05], (**B**) inhibition of FP-BRC-R2 growth by CEO [F (7, 16) = 391.72, *p* < 0.05] and Ne-CEO [F (4, 10) = 194.95, *p* < 0.05], (**C**) inhibition of FB_1_ and FB_2_ by CEO [F (6, 14) = 264.46, *p* < 0.05] [F (5, 12) = 273.14, *p* < 0.05] and Ne-CEO (F (3, 8) = 473.46, *p* < 0.05] [F (3, 8) = 571.49, *p* < 0.05], (**D**) effect of Ne-Cs on inhibition of FP-BRC-R2 growth [F (6, 14) = 178.93, *p* < 0.05] and FB_1_ (F (5, 12) = 151.36, *p* < 0.05], FB_2_ [F (5, 12) = 202.73, *p* < 0.05] production, (**E**) fungitoxic spectrum of CEO and Ne-CEO [F (8, 18) = 6.16, *p* < 0.05] against different rice contaminating fungi. Note: Values are mean (n = 3) ± SE; different letters represent significant differences at *p* value < 0.05 according to ANOVA and Tukey’s multiple comparison tests.

The CEO release was observed in three different phases in PBS medium (Figure 6A). Initial burst delivery of CEO with 20.13%, 15.22%, and 12.96% release was recorded in the beginning within (0–4) h, (4–8) h, and (8–12) h, which was mainly due to the instant diffusion of CEO adhered at the particle surface [17]. Thereafter, steady rate release was found between (12–24) h, (24–48) h, and (48–72) h. This gradual release of CEO has been explained by steady rate diffusion of CEO from chitosan polymeric matrix to PBS. At last, stationary phase suggested very slow release of CEO from chitosan matrix, (72–168) h, because of change in glossy polymer to rubbery state after interaction with CEO and PBS medium. Our result is in line with the investigation of Soltanzadeh et al. [54] with three-step release of *Cymbopogon commutatus* essential oil from chitosan nanoparticles in phosphate and acetate buffer. The sustained release pattern of CEO in emulsion system suggests the propensity for greater protection of stored rice samples after nanoemulsion fumigation.

### 3.6. Antifungal and Anti-Fumonisin Effectiveness of CEO and Ne-CEO: In Vitro Study

The antifungal and anti-fumonisin effectiveness of CEO and Ne-CEO is reported in Figure 6B,C. CEO displayed significant (*p* < 0.05, df = 7, 16, F = 391.72) inhibition of FP-BRC-R2 growth and minimum inhibitory concentration (MIC) was recorded at 0.7 µL/mL (Figure 6B). The CEO completely suppressed the FB_1_ and FB_2_ biosynthesis at 0.6 and 0.5 µL/mL, respectively (Figure 6C). Our result displayed superiority over the clove, cinnamon, oregano, lemongrass, and palmarose essential oils for the inhibition of FP-BRC-R2 growth and FB_1_ production [55]. The Ne-CEO showed better antifungal activity against FP-BRC-R2 at 0.4 µL/mL (Figure 6B). Interestingly, 100% suppression of FB_1_ and FB_2_ production was achieved by Ne-CEO at 0.3 µL/mL (Figure 6C). The Ne-Cs showed very little antifungal activity and completely inhibited FP-BRC-R2 growth and FB_1_, FB_2_ biosynthesis at 6.0 and 5.0 µL/mL, respectively (Figure 6D). Better performance of Ne-CEO for anti-fumonisin activity over antifungal activity may be related to the binding of essential oil-loaded nanoemulsionic particles with functional moieties of FB_1_ and FB_2_. Moreover, the controlled and sustained release of CEO with better cellular penetration capacity may enhance the interaction with cellular components resulting in superior antifungal and anti-fumonisin activity of Ne-CEO over unencapsulated CEO [56]. Most importantly, both the CEO and Ne-CEO showed broad spectrum fungitoxicity against *F. verticillioides*, *Aspergillus flavus*, *A. niger*, *A. repens*, *Penicillium italicum*, *Alternaria alternata*, *A. humicola*, and *Curvularia lunata* at their respective MIC dose, i.e., 0.7 and 0.4 µL/mL, respectively (Figure 6E).

### 3.7. Mechanisms Related to Antifungal and Anti-Fumonisin Activity

**Figure 7:**
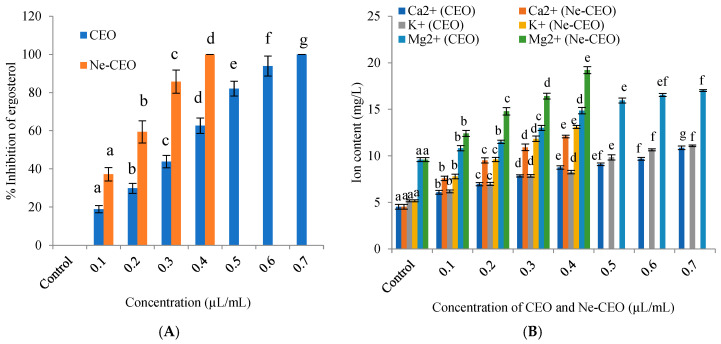

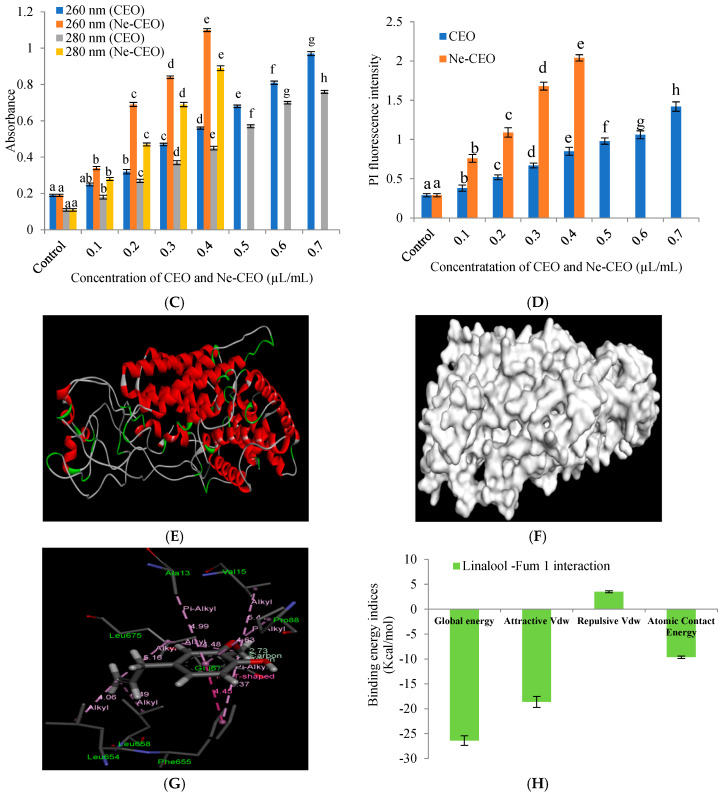
(**A**) Inhibition of ergosterol content in FP-BRC-R2 cells by CEO [F (7, 16) = 303.70, *p* < 0.05] and Ne-CEO [F (4, 10) = 216.47, *p* < 0.05], (**B**) effect of CEO and Ne-CEO on leakage of Ca^2+^ [F (7, 16) = 131.58, *p* < 0.05], K^+^ [F (7, 16) = 155.42, *p* < 0.05], and Mg^2+^ [F (7, 16) = 129.76, *p* < 0.05] ions from FP-BRC-R2 cells, (**C**) effect of CEO and Ne-CEO on efflux of 260 nm [F (7, 16) = 89.83, *p* < 0.05] and 280 nm [F (4, 10) = 75.22, *p* < 0.05] absorbing materials from FP-BRC-R2 cells, (**D**) effect of CEO [F (7, 16) = 124.07, *p* < 0.05] and Ne-CEO [F (4, 10) = 170, *p* < 0.05] on propidium iodide (PI) fluorescence intensity in FP-BRC-R2 cells, (**E**,**F**) structure of Fum 1 protein, (**G**) binding of linalool with Fum 1 protein, (**H**) binding energy indices of linalool with Fum 1 protein. Note: Values are mean (n = 3) ± SE; different letters represent significant differences at *p* value < 0.05 according to ANOVA and Tukey’s multiple comparison tests.

Ergosterol is an essential sterol molecule in fungi required for plasma membrane integrity and stability, and also participates in cell growth, division, and membrane fluidity [57]. It also regulates fungal metabolism and adaptation to the external environment. As shown in Figure 7A, the CEO treatment at different doses significantly (*p* < 0.05, df = 7, 16, F = 303.70) reduced the ergosterol content of FP-BRC-R2 cells and completely suppressed at 0.7 µL/mL. This reduction in ergosterol content validates that CEO inhibits the viability of FP-BRC-R2 cells. Remarkable obstruction of ergosterol production by CEO could be assigned to reduced activity of lanosterol-14-α demethylase (CYP 51) enzyme which is responsible for oxidative removal of 14-α-methyl group from lanosterol, a crucial step for ergosterol synthesis [58]. The compensatory effect of ergosterol production might be responsible for the occurrence of hydroxyl groups in essential oils [59]. The current finding coincides with the outcome of Wei et al. [60] for impediment in ergosterol production in *F. sambucinum* by cinnamaldehyde. Interestingly, the Ne-CEO showed greater potentiality to inhibit the production of ergosterol at lower doses (Figure 7A). The decreased production of ergosterol by Ne-CEO as compared to CEO has been associated with nano-range diameter of particles having greater surface/volume ratio, sustained delivery, and better inhibition kinetics.

The treatment of CEO at different doses caused leakage of Mg^2+^, K^+^, and Ca^2+^ ions from FP-BRC-R2 cells. The CEO also showed the outflow of 260 nm (nucleic acids) and 280 nm (proteins) absorbing materials from FP-BRC-R2 cells. However, the Ne-CEO treatment induced the leakage of Mg^2+^, K^+^, Ca^2+^ ions and nucleic acids, and proteins (Figure 7B,C). Excessive leakage of vital cellular constituents by Ne-CEO resulted in perturbation in maintenance of osmotic pressure of cells and may lead to cell death [61].

The plasma membrane integrity was determined by the ability of PI to penetrate the cells and bind with cellular DNA following reinforcement of the fluorescence intensity with compromised plasma membrane [62]. At different doses of CEO, the PI fluorescence intensity was found to significantly (*p* < 0.05, df = 7, 16, F = 124.07) increase in FP-BRC-R2 cells, while the Ne-CEO treatment showed greater fluorescence intensity as compared to the unencapsulated CEO treatment (Figure 7D). Hence, Ne-CEO treatment was found more effective in destabilizing the plasma membrane of FP-BRC-R2 cells that might be due to formation of more lesions. Our findings are in harmony with the investigation of Rani et al. [63] for increase in PI fluorescence intensity in *Aspergillus foetidus* cells by *Monarda citriodora* essential oil. All the findings of the current investigation confirmed the loss in plasma membrane integrity as well as permeability in FP-BRC-R2 cells by CEO and Ne-CEO treatment, hence suggesting plasma membrane as the target site of antifungal activity.

The anti-fumonisin mechanism of action was investigated through in silico interaction of linalool with Fum 1 protein. The Fum 1 is a polyketide synthase protein which catalyzes the linear polyketide of fumonisins [64]. The structure of Fum 1 protein is presented in Figure 7E,F. The molecular interaction was analyzed through atomic contact energy, global energy, and attractive and repulsive Van der Waals forces (Figure 7H). The energy modeling validated different hydrogen bond-mediated interaction of linalool with Pro 88, Val 15, Leu 675, and Ala 13 amino acids of Fum 1 protein (Figure 7G). The highest affinity of test compounds against Fum 1 has been associated with more negative binding energy. The present result is in agreement with the report of Murugan et al. [65] for inhibition of aflatoxin B_1_ by interaction of methyl palmitate and phenylquinazoline-4-carboximidamide with Ver 1 protein. Conclusively, the strong interaction of linalool with Fum 1 protein led to functional changes or stereo-specific binding at the catalytic site resulting in inhibition of fumonisins biosynthesis.

### 3.8. Radical Neutralizing Capacity of CEO and Ne-CEO

In addition to fungal association and fumonisin contamination, oxidative stress to foods impairs the cell function, leading to toxicity and development of different disease symptoms [66]. Oxidative stress is mainly referring to the imbalance between antioxidant and reactive oxygen species (ROS). The ROS production facilitates the damage of food macromolecules. The presence of external stimuli like fumonisins may disrupt the redox environment in cells and enhance the production of ROS [67]. Hence, the assessment of radical neutralizing capacity of CEO and Ne-CEO by DPPH and ABTS analysis is an important part of the study to scavenge the ROS molecules. The intensity of antioxidant activity was measured in terms of IC_50_ value, meaning the half maximal concentration to inhibit the free radicals. The IC_50_ value of CEO against DPPH and ABTS analysis was recognized as 15.87 and 3.01 µL/mL, respectively. High value of antioxidant capacity could be attributed to different phenols in CEO that are responsible for donating H atom to phenolic hydroxyl followed by further stabilization of π electrons of aromatic rings by resonance [68]. The Ne-CEO showed better antioxidant activity at 11.04 and 2.21 µL/mL against DPPH and ABTS free radicals, respectively. Better performance of Ne-CEO to neutralize the free radicals has been linked with synergism between chitosan and CEO molecules with nanoemulsionic droplets (greater surface-area-to-volume ratio)-mediated sustained release at the target site. Improvement in radical neutralization potency of *Lippia origanoides* essential oil after encapsulation in chitosan and p-coumaric acid has also been reported by Damasceno et al. [69]. Superior free radical neutralizing activity of Ne-CEO may be helpful for preserving the foods in place to harmful synthetic antioxidants.

### 3.9. In Situ Antifungal and Anti-Fumonisin Efficacy of CEO and Ne-CEO in Stored Rice

**Figure 8:**
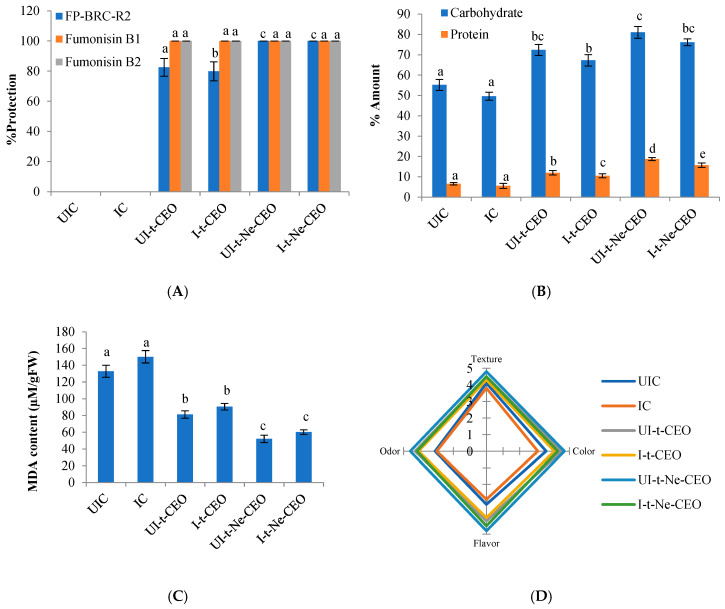
(**A**) In situ protection of rice samples against FP-BRC-R2 growth, and FB_1_ and FB_2_ production [F (5, 12) = 773.39, *p* < 0.05], (**B**) effect of CEO and Ne-CEO on carbohydrate [F (5, 12) = 23.03, *p* < 0.05] and protein [F (5, 12) = 207.07, *p* < 0.05] content of rice samples, (**C**) effect of CEO and Ne-CEO on lipid peroxidation [F (5, 12) = 56.32, *p* < 0.05] of rice samples, (**D**) effect of CEO and Ne-CEO on organoleptic properties of rice. Note: Values are mean (n = 3) ± SE; different letters represent significant differences at *p* value < 0.05 according to ANOVA and Tukey’s multiple comparison tests.

In situ efficacy of CEO and Ne-CEO was investigated in rice samples (Gobindobhog variety) for 6 months of storage. The protection of rice samples against FP-BRC-R2 was found to be 79.87 and 82.52% at inoculated and uninoculated treatment sets at MIC dose of CEO (0.7 µL/mL). However, the Ne-CEO treatment at 0.4 µL/mL (MIC dose) caused 100% inhibition of FP-BRC-R2 infestation in inoculated and uninoculated rice samples. Most importantly, the CEO and Ne-CEO at MIC dose displayed complete suppression of FB_1_ and FB_2_ production in rice samples (Figure 8A). The FB_1_ concentration in inoculated and uninoculated control rice samples was recognized as 12.03 and 10.97 µg/kg while FB_2_ content was reported as 8.63 and 7.19 µg/kg, respectively. During in vitro investigation, the unencapsulated CEO completely suppressed the fungal growth at MIC dose; however, at the time of in situ investigation, the CEO was not able to completely inhibit FP-BRC-R2 growth in real food systems (rice samples) which may be due to absorbance of some CEO components by the rice grains itself [70]. In case of Ne-CEO treatment, 100% fungal inhibition was achieved due to controlled and sustained delivery of CEO from chitosan nanobiopolymer. The absolute suppression of FB_1_ and FB_2_ has been associated with good antioxidant capacity along with improvement in radical quenching capacity of CEO by encapsulation and controlled release at the location where mycelial invasion easily occurred. Our result is similar to the findings of Singh et al. [43], validating in situ protection of millet samples against *A. flavus* and aflatoxin B_1_ contamination by nanoencapsulated *Ocimum americanum* essential oil after 1 year of storage. However, the finding of the current study shows superiority in terms of 100% inhibition of FP-BRC-R2 and FB_1_, FB_2_ contamination in rice samples after 6 months of storage, demonstrating the potential of Ne-CEO as a suitable fungicide in agricultural store houses.

### 3.10. Effect of CEO and Ne-CEO on Carbohydrate, Protein Content, and Lipid Peroxidation in Rice

Carbohydrates and proteins are important energy-providing components in rice grains. The percent amount of carbohydrate and protein content in uninoculated and inoculated control rice samples was found to be 55.19, 49.63, and 6.58, 5.59%, respectively. Significant reduction in percent amount of carbohydrate (*p* < 0.05, df = 5, 12, F = 23.03) and protein content (*p* < 0.05, df = 5, 12, F = 207.07) has been linked with fumonisins-mediated oxidative deterioration in control rice samples. In case of CEO-fumigated rice samples, the percent amount of carbohydrate and protein content has been observed as 72.41, 67.29, and 12.01, 10.53%, respectively. Interestingly, the Ne-CEO showed better performance in preventing the carbohydrate and protein loss in treated rice samples for up to 6 months of storage (Figure 8B). The protection may be associated with promising antioxidant capacity of CEO and Ne-CEO offering inhibition of free radicals-mediated biodeterioration of carbohydrate and protein content. In addition to fumonisin contamination, assessing the peroxidation of rice lipids during storage is of great interest. It is a common phenomenon that occurs in oxidation-prone food systems resulting in off-taste, off-flavor, and reduction in nutritional contents [71]. The extent of lipid peroxidation is measured by means of MDA because it is a common reactive aldehyde byproduct and used as potent marker for assessing the oxidative stress [72]. The obtained result revealed high concentration of MDA in inoculated (150.19 µM/g FW) and uninoculated rice (132.86 µM/g FW) samples (Figure 8C). The CEO showed significant (*p* < 0.05, df = 5, 12, F = 56.32) retardation of MDA content over 6 months of storage. However, the Ne-CEO displayed better potentiality to reduce the MDA content in rice samples by up to 60.18 µM/g FW. Better retardation for peroxidation of rice lipids has been linked with the inhibition of free radicals chain reaction by nanometric particles of Ne-CEO, with greater surface/volume ratio and controlled as well as targeted delivery of CEO in fumigated food system. The current finding coincides with the previous investigation of Deepika et al. [73] who reported the reduction in MDA content in chia seeds by nanoencapsulated *Petroselinum crispum* essential oil.

### 3.11. Effect of CEO and Ne-CEO on Organoleptic Properties of Rice

Effect of CEO and Ne-CEO on odor, color, flavor, and texture of rice samples was determined by a hedonic scale test reflecting the willingness of consumers for consumption purposes (Figure 8D). The scores for color, odor, flavor, and texture of uninoculated and inoculated control rice samples that were significantly reduced and found unacceptable was mainly attributed to lipid peroxidation and free radical generation. The treatment of rice samples with CEO at MIC dose showed higher scores for color, odor, flavor, and texture as compared to control rice samples. Rice samples fumigated with Ne-CEO at MIC dose showed better scores ranging between 4 and 5 for all the organoleptic attributes. The sustained release of CEO from chitosan matrix with targeted action may be a possible reason for prolonged preservation of organoleptic properties of rice. The findings of the current study align with the report of Chaudhari et al. [24] for satisfactory scores of maize samples after fumigation with nanoencapsulated *Origanum majorana* essential oil.

### 3.12. Phytotoxicity Assay in Rice Seeds

The CEO and Ne-CEO fumigated rice samples did not show any phytotoxic effect, demonstrating no loss in viability of seeds. The uninoculated and inoculated control rice seeds displayed somewhat reduced lengths of plumule and radicle, which may be associated with the loss of carbohydrate and protein content, as recognized during the in situ analysis (Table 6A–C). However, the non-phytotoxic nature of Ne-CEO may recommend the utilization of rice seeds for further sowing and intended agricultural practices in the next growing season.

## 4. Conclusions

Encapsulation of *Coriandrum sativum* essential oil in chitosan polymer by ionic gelation showed improvement in antifungal and anti-fumonisin activity. The biochemical mechanism of Ne-CEO against *F. proliferatum* was associated with depletion of ergosterol content, leakage of cellular cations, nucleic acids, proteins, and destruction of plasma membrane integrity. The underlying anti-fumonisin mechanism of action was linked with in silico interaction of linalool with Fum 1 protein. Further, during the in situ investigation, the Ne-CEO exhibited remarkable efficacy in protecting rice samples against fungal proliferation, fumonisins-mediated nutritional deterioration, and lipid peroxidation. In addition, the Ne-CEO did not show any phytotoxic effect in seed germination and alteration in organoleptic properties of rice samples, demonstrating its potentiality to be utilized as nano-green food preservative in agricultural and food industries.

## Figures and Tables

**Table 1:** Moisture content and pH of different rice varieties.

Rice Varieties	Moisture Content (%)	pH
Gobindobhog	11.96 ± 0.18 ^a^	6.46 ± 0.13 ^ab^
Boro-minikit	10.52 ± 0.20 ^c^	6.23 ± 0.20 ^bc^
Boro-1153-jath	10.89 ± 0.11 ^bc^	6.87 ± 0.18 ^a^
Barshar-CM	9.85 ± 0.17 ^d^	6.08 ± 0.92 ^c^
Swarna dhan	11.26 ± 0.25 ^ab^	6.37 ± 0.11 ^bc^
Radhunipagol	10.57 ± 0.30 ^c^	6.22 ± 0.68 ^bc^
ANOVA test parameters		
*p* value	<0.05	<0.05
F value	19.91	7.23
df	5, 12	5, 12

Note: Values are mean (n =3) ± SE; different superscript letters within the same column indicate statistically significant differences (*p* < 0.05) as determined by one-way ANOVA followed by Tukey’s multiple comparison test. Groups sharing the same letter are not significantly different from each other.

**Table 2:** Mycobiota analysis of different rice varieties.

Rice Varieties	Fungal Species									Total No. of Species	Occurrence Frequency (%)
	*F.p*	*F.v*	*A.f*	*A.n*	*A.r*	*P.i*	*A.a*	*C.l*	*A.h*		
Gobindobhog	21	20	12	8	9	4	3	2	1	9	22.59
Boro-minikit	18	24	16	5	7	2	-	-	-	6	20.33
Boro-1153-jath	14	20	6	9	-	-	2	1	2	7	15.25
Barshar-CM	10	8	5	-	-	2	1	4	1	7	8.75
Swarna dhan	29	23	6	3	4	-	3	-	-	6	19.20
Radhunipagol	16	19	-	-	5	4	-	2	3	6	13.84
Total colony of each fungus	108	114	45	25	25	12	9	9	7		
Relative density (%)	30.50	32.20	12.71	7.06	7.06	3.38	2.54	2.54	1.97		

Note: - = Not found, *F.p* = *Fusarium proliferatum*, *F.v* = *Fusarium verticillioides*, *A.f* = *Aspergillus flavus*, *A.n* = *Aspergillus niger*, *A.r* = *Aspergillus repens*, *P.i* = *Penicillium italicum*, *A.a* = *Alternaria alternata*, *C.l* = *Curvularia lunata*, *A.h* = *Alternaria humicola*.

**Table 3:** FB_1_ and FB_2_ content of different isolates of *F. proliferatum* in rice samples.

Rice Samples	Isolates of *F. proliferatum*	FB_1_ Content (µg/L)	FB_2_ Content (µg/L)
Gobindobhog	FP-BRC-R1	87.63	80.34
	**FP-BRC-R2**	**142.20**	**135.22**
	FP-BRC-R3	-	-
	FP-BRC-R4	102.49	95.62
	FP-BRC-R5	84.25	80.19
	FP-BRC-R6	62.11	57.21
	FP-BRC-R7	47.52	40.88
Boro-minikit	FP-BRC-R8	95.03	90.32
	FP-BRC-R9	87.22	82.43
	FP-BRC-R10	110.54	96.85
	FP-BRC-R11	50.74	47.20
	FP-BRC-R12	23.14	19.85
	FP-BRC-R13	43.97	36.55
	FP-BRC-R14	76.31	68.26
Boro-1153-jath	FP-BRC-R15	-	-
	FP-BRC-R16	-	-
	FP-BRC-R17	49.86	45.23
	FP-BRC-R18	130.64	124.95
	FP-BRC-R19	46.20	42.29
	FP-BRC-R20	84.77	80.64
	FP-BRC-R21	29.36	25.41
Barshar-CM	FP-BRC-R22	59.53	52.34
	FP-BRC-R23	73.01	70.19
	FP-BRC-R24	89.56	82.43
	FP-BRC-R25	39.54	30.99
	FP-BRC-R26	73.06	70.54
	FP-BRC-R27	85.46	82.06
	FP-BRC-R28	76.21	72.31
Swarna dhan	FP-BRC-R29	58.19	50.46
	FP-BRC-R30	72.03	70.22
	FP-BRC-R31	54.22	48.62
	FP-BRC-R32	61.97	57.22
	FP-BRC-R34	138.02	130.89
	FP-BRC-R35	49.20	45.20
	FP-BRC-R36	65.88	62.41
Radhunipagol	FP-BRC-R41	79.06	74.65
	FP-BRC-R42	43.06	41.33
	FP-BRC-R43	86.34	82.09
	FP-BRC-R44	95.20	92.43
	FP-BRC-R45	124.62	120.85
	FP-BRC-R47	72.16	70.31
	FP-BRC-R48	34.92	31.64

Note: Bold represents the most toxigenic strain of *Fusarium proliferatum*.

**Table 4:** GC-MS analysis of *Coriandrum sativum* essential oil.

S No.	Component	Retention Time (min)	% Availability	Retention Index
1.	α-pinene	5.02	2.87	932
2.	1-octanal (n-octanal)	6.31	0.84	998
3.	O-cymene	7.09	0.38	1022
4.	Limonene	7.85	0.49	1024
5.	Terpinolene	8.27	0.97	1086
6.	**Linalool**	**8.85**	**66.46**	1095
7.	Citronellal	10.67	1.06	1148
8.	α-terpineol	11.15	1.22	1186
9.	Methyl chavicol	12.21	0.94	1195
10.	Z-anethole	12.79	2.01	1249
11.	Undecanal	16.56	1.85	1305
12.	**Geranyl acetate**	**19.81**	**14.22**	1379
13.	Dodecanal	22.77	0.17	1408
14.	2E-dodecanal	27.89	0.21	1464
15.	γ-Asarone	29.40	1.03	1572
		Total	94.72	

Note: Bold represents the major compounds.

**Table 5:** Encapsulation potency and loading potency of Ne-CEO.

Chitosan/CEO Ratio (*w*/*v*)	LP (%)	EP (%)
1:0.0	0.00 ± 0.00 ^a^	0.00 ± 0.00 ^a^
1:0.2	0.82 ± 0.18 ^b^	29.63 ± 2.06 ^b^
1:0.4	1.63 ± 0.52 ^c^	52.87 ± 1.98 ^c^
1:0.6	3.89 ± 0.46 ^d^	72.19 ± 2.96 ^d^
1:0.8	5.04 ± 0.26 ^d^	92.11 ± 3.72 ^d^
1:1	4.10 ± 0.34 ^e^	80.63 ± 4.63 ^e^
ANOVA test parameters		
*p* value	<0.05	<0.05
F value	137.37	39.75
df	5, 12	5, 12

Note: Values are mean (n = 3) ± SE; different superscript letters within the same column indicate statistically significant differences (*p* < 0.05) as determined by one-way ANOVA followed by Tukey’s multiple comparison test. Groups sharing the same letter are not significantly different from each other.

**Table 6:** (**A**) Seed germination of UIC and IC rice samples. (**B**) Seed germination of UI-t-CEO and I-t-CEO rice samples. (**C**) Seed germination of UI-t-Ne-CEO and I-t-Ne-CEO rice samples.

(**A**)					
**Duration of Exposure (h)**	**Mean Length of Seedling (cm)**		**Duration of Exposure (h)**	**Mean Length of Seedling (cm)**	
	**UIC**			**IC**	
	**Plumule**	**Radicle**		**Plumule**	**Radicle**
24	0.11 ± 0.02 ^a^	0.59 ± 0.07 ^a^	24	0.08 ± 0.02 ^a^	0.46 ± 0.04 ^a^
48	0.21 ± 0.04 ^a^	0.84 ± 0.06 ^a^	48	0.12 ± 0.06 ^a^	0.72 ± 0.05 ^b^
72	0.43 ± 0.05 ^b^	1.25 ± 0.12 ^b^	72	0.35 ± 0.04 ^b^	0.81 ± 0.06 ^b^
96	0.62 ± 0.04 ^c^	1.69 ± 0.06 ^c^	96	0.50 ± 0.04 ^b^	1.20 ± 0.12 ^c^
120	0.86 ± 0.05 ^d^	1.89 ± 0.09 ^c^	120	0.65 ± 0.05 ^e^	1.54 ± 0.18 ^d^
ANOVA test parameters			ANOVA test parameters		
*p* value	<0.05	<0.05	*p* value	<0.05	<0.05
F value	47.79	47.31	F value	35.34	35.85
df	4, 10	4, 10		4, 10	4, 10
(**B**)					
**Duration of Exposure (h)**	**Mean Length of Seedling (cm)**		**Duration of Exposure (h)**	**Mean Length of Seedling (cm)**	
	**UI-t-CEO**			**I-t-CEO**	
	**Plumule**	**Radicle**		**Plumule**	**Radicle**
24	0.19 ± 0.06 ^a^	0.61 ± 0.07 ^a^	24	0.15 ± 0.03 ^a^	0.58 ± 0.07 ^a^
48	0.40 ± 0.05 ^a^	0.97 ± 0.07 ^ab^	48	0.37 ± 0.05 ^b^	0.89 ± 0.05 ^b^
72	0.68 ± 0.06 ^b^	1.35 ± 0.19 ^b^	72	0.63 ± 0.06 ^c^	1.28 ± 0.15 ^c^
96	0.96 ± 0.06 ^c^	2.27 ± 0.15 ^c^	96	0.89 ± 0.04 ^d^	2.09 ± 0.13 ^d^
120	1.11 ± 0.04 ^c^	2.51 ± 0.31 ^c^	120	1.02 ± 0.05 ^d^	2.55 ± 0.07 ^e^
ANOVA test parameters			ANOVA test parameters		
*p* value	<0.05	<0.05	*p* value	<0.05	<0.05
F value	47.56	32.81	F value	51.95	64.51
df	4, 10	4, 10	df	4, 10	4, 10
(**C**)					
**Duration of Exposure (h)**	**Mean Length of Seedling (cm)**		**Duration of Exposure (h)**	**Mean Length of Seedling (cm)**	
	**UI-t-Ne-CEO**			**I-t-Ne-CEO**	
	**Plumule**	**Radicle**		**Plumule**	**Radicle**
24	0.23 ± 0.06 ^a^	0.69 ± 0.11 ^a^	24	0.20 ± 0.05 ^a^	0.65 ± 0.08 ^a^
48	0.51 ± 0.07 ^b^	1.23 ± 0.13 ^b^	48	0.45 ± 0.06 ^b^	1.15 ± 0.06 ^ab^
72	0.88 ± 0.05 ^c^	1.65 ± 0.09 ^b^	72	0.80 ± 0.05 ^c^	1.39 ± 0.17 ^b^
96	1.06 ± 0.04 ^cd^	2.52 ± 0.21 ^c^	96	0.95 ± 0.05 ^cd^	2.23 ± 0.14 ^c^
120	1.21 ± 0.03 ^d^	2.95 ± 0.06 ^c^	120	1.16 ± 0.08 ^d^	2.87 ± 0.12 ^d^
ANOVA test parameters			ANOVA test parameters		
*p* value	<0.05	<0.05	*p* value	<0.05	<0.05
F value	47.48	49.63	F value	49.67	46.33
df	4, 10	4, 10	df	4, 10	4, 10

Note: Values are mean (n = 3) ± SE; different superscript letters within the same column indicate statistically significant differences (*p* < 0.05) as determined by one-way ANOVA followed by Tukey’s multiple comparison test. Groups sharing the same letter are not significantly different from each other.

## Data Availability

The original contributions presented in this study are included in the article. Further inquiries can be directed to the corresponding author.
